# β-Adrenoceptor Agonists Attenuate Thrombin-Induced Impairment of Human Lung Endothelial Cell Barrier Function and Protect the Lung Vascular Barrier during Resuscitation from Hemorrhagic Shock

**DOI:** 10.3390/biomedicines12081813

**Published:** 2024-08-09

**Authors:** Michelle Y. McGee, Ololade Ogunsina, Sadia N. Boshra, Xianlong Gao, Matthias Majetschak

**Affiliations:** 1 Department of Surgery, University of South Florida Morsani College of Medicine, Tampa, FL 33612, USA; mcgeem@usf.edu (M.Y.M.); oogunsina@usf.edu (O.O.); sadianoosratboshra@usf.edu (S.N.B.); xgao1@usf.edu (X.G.); 2 Department of Chemistry, University of South Florida, Tampa, FL 33612, USA; 3 Department of Molecular Pharmacology & Physiology, University of South Florida, Tampa, FL 33612, USA

**Keywords:** adrenoceptor agonists, lung vascular barrier function, thrombin, Evans blue extravasation, β-adrenergic receptors

## Abstract

β-adrenoceptor (β-AR) agonists are known to antagonize thrombin-induced impairment (TII) of bovine and ovine lung endothelial barrier function. The effects of adrenoceptor agonists and other vasoactive agents on human lung microvascular endothelial cell (HULEC-5a) barrier function upon thrombin exposure have not been studied. Furthermore, it is unknown whether the in vitro effects of adrenoceptor agonists translate to lung protective effects in vivo. We observed that epinephrine, norepinephrine, and phenylephrine enhanced normal and prevented TII of HULEC-5a barrier function. Arginine vasopressin and angiotensin II were ineffective. α_1B_-, α_2A/B_-, and β_1/2_-ARs were detectable in HULEC-5a by RT-PCR. Propranolol but not doxazosin blocked the effects of all adrenoceptor agonists. Phenylephrine stimulated β_2_-AR-mediated Gαs activation with 13-fold lower potency than epinephrine. The EC_50_ to inhibit TII of HULEC-5a barrier function was 1.8 ± 1.9 nM for epinephrine and >100 nM for phenylephrine. After hemorrhagic shock and fluid resuscitation in rats, Evans blue extravasation into the lung increased threefold (*p* < 0.01 vs. sham). Single low-dose (1.8 μg/kg) epinephrine administration at the beginning of resuscitation had no effects on blood pressure and reduced Evans blue extravasation by 60% (*p* < 0.05 vs. vehicle). Our findings confirm the effects of β-adrenoceptor agonists in HULEC-5a and suggest that low-dose β-adrenoceptor agonist treatment protects lung vascular barrier function after traumatic hemorrhagic shock.

## 1. Introduction

The impairment of endothelial barrier function in infectious and non-infectious disease processes, such as traumatic hemorrhagic shock, sepsis, and burns, leads to capillary leakage, third spacing of fluids into tissues, and edema formation, which are hallmarks in the etiology of acute respiratory distress syndrome (ARDS) [1]. In these patients, vasoactive blood pressure-increasing drugs, of which catecholamines, arginine vasopressin (AVP), and angiotensin II (ATII) are approved by the Federal Drug Administration [2,3], are often required to stabilize blood pressure and hemodynamics.

Several lines of evidence suggest that catecholamines, besides their effects on hemodynamics, enhance lung endothelial cell barrier function under various experimental conditions. β-adrenoceptor agonists have been described to antagonize thrombin-induced impairment of endothelial cell barrier function in the bovine pulmonary artery endothelial cell line CCL 209 in vitro and to attenuate increases in pulmonary transvascular fluid and protein exchange after the infusion of thrombin in sheep [4,5,6,7]. Because thrombin-mediated activation of protease-activated receptors on lung endothelial cells has been recognized as an important pathophysiological mechanism leading to impaired lung vascular barrier function in ARDS [8,9,10], the observation that adrenoceptor agonists inhibit thrombin-induced effects on lung endothelial barrier function may suggest that adrenoceptor agonists could be used to attenuate lung injury and ARDS development in patients. However, the effects of adrenoceptor agonists on thrombin-induced impairment of human lung microvascular endothelial cell barrier function have not been tested. Moreover, it is unknown whether AVP and angiotensin II may also modulate thrombin-mediated impairment of human lung endothelial barrier function and whether the effects of adrenoceptor agonists may translate to lung protective effects in disease models in vivo.

Thus, in the present study, we employed the human lung microvascular endothelial cell line HULEC-5a to evaluate the effects of adrenoceptor agonists, AVP, and ATII on normal and thrombin-stimulated endothelial cell barrier function in vitro. Furthermore, we utilized a rat model of hemorrhagic shock and fluid resuscitation, which leads to the impairment of lung vascular barrier function [11], to assess whether observed in vitro effects can be recapitulated in vivo. 

## 2. Materials and Methods

### 2.1. Reagents

Epinephrine, norepinephrine, phenylephrine, propranolol, doxazosin, arginine vasopressin, angiotensin II, thrombin, and fluorescein isothiocyanate (FITC)-dextran were purchased from Sigma-Aldrich, St. Louis, MO, USA.

### 2.2. Cells and Cell Culture

HULEC-5a (human lung microvascular endothelial) cells were purchased from ATCC, Manassas, VA, USA (CRL-3244). HULEC-5a cells were cultured in vascular basal cell media (PCS-100-030, ATCC) with the addition of supplemental growth factors (endothelial cell growth kit—VEGF PCS-100-041, ATCC), 100 U/mL penicillin, and 100 μg/mL streptomycin at 37 °C, 5% CO_2_ until they reached >90% confluence, and they were used at passages 3–7 after thawing from liquid nitrogen. Growth media were aspirated, and cells were trypsinized with 0.25% Trypsin & 0.53 mM EDTA (ATCC 30-2101) for 3–4 min or until all cells detached. Trypsinization was stopped using 4 mL of neutralization buffer (5% FBS in PBS), and the cells were centrifuged for 5 min at 250× *g*. The supernatant was removed, and the cells were resuspended in 1 mL endothelial medium, counted, and diluted to 0.5 × 10^6^ cells/mL. 

### 2.3. In Vitro Endothelial Cell Permeability Assays

Permeability assays were performed using the 96-well In Vitro Vascular Permeability Assay Kit (Millipore, Lakeland, FL, USA, ECM642), as described previously [12,13,14]. In brief, inserts (top wells) were hydrated with 135 μL of endothelial media and incubated for 15 min. A quantity of 100 μL of media was removed from each insert and replaced with 100 μL of a HULEC-5a cell suspension (0.5 × 10^5^ cells/insert). Then, 250 μL of endothelial media was added to the bottom well via a basolateral access hole at the top right of each insert. The plate was covered and incubated at 37 °C/5% CO_2_ for 48 h to form a confluent monolayer. After 48 h, the insert was placed onto a new bottom well plate. All fluid was removed from the top well and immediately replaced with FITC-dextran in a 1:50 dilution plus vehicle (growth medium only) or various combinations of drugs in the presence or absence of 25 nM thrombin, and the bottom wells were filled as described before. After 1 h and 4 h, the top insert was removed, and 100 μL of liquid was sampled from the bottom wells. Relative fluorescence units were then measured in a microplate reader (Cytation 1; BioTek Instruments, Winooski, VT, USA) at λ_excitation/emission_ 485/528 nm.

### 2.4. Reverse Transcription Polymerase Chain Reaction (RT-PCR)

Total RNA was extracted from cells using TRIzol from Invitrogen (Waltham, MA, USA). RNA was reverse transcribed to cDNAs with the Applied Biosystems High-Capacity cDNA Reverse Transcription Kit (Thermo Fisher Scientific, Waltham, MA, USA) following the manufacturer’s manual. Human adrenoceptor primers were synthesized by Integrated DNA Technologies (Coralville, IA, USA). Reverse transcription PCR was run with 50 ng cDNA for 32 cycles and PCR products were separated on a 1.5% agarose gel.

### 2.5. Gαs Activation Assay

Gαs activation was measured using the TRUPATH bioluminescence resonance energy tranfer (BRET) biosensor method, as described [15,16,17,18]. HEK293T cells were plated in a 6-well plate and transfected with TRUPATH biosensor plasmids (Gαs-Rluc8 plus Gβ3 and Gγ9-GFP2) together with β_2_-AR. One day after transfection, cells were trypsinized and replated to 96-well poly-L-lysine precoated plates. After overnight incubation, cells were replaced with 0.1% glucose/PBS. Coelenterazine 400a in a final concentration of 5 μM was added to cells and incubated at room temperature for 3 min. Epinephrine or phenylephrine at various concentrations was added to cells and incubated at room temperature for 3 min before luminescence was measured at 410 and 515 nm. The BRET signal was calculated as the ratio of the relative luminescence signals (RLUs) measured at 515 nm over RLUs measured at 410 nm. The BRET changes were calculated by subtracting the BRET signal of untreated cells.

### 2.6. Hemorrhagic Shock and Fluid Resuscitation Model

All procedures followed the National Institutes of Health Guidelines for Use of Laboratory Animals and were approved by the Institutional Animal Care and Use Committee of the University of South Florida (IS00012148). Male Sprague Dawley rats (300–350 g) were purchased from Envigo (Indianapolis, IN, USA).

Anesthesia induction was performed by placing the animal in a bell jar with isoflurane-soaked gauze. After the animal was appropriately anesthetized, it was moved to the operative field and exposed to continuous 2.6% isoflurane anesthesia via a nosecone administered by the SomnoSuite small animal anesthesia system (Kent Scientific Corporation, Torrington, CT, USA). The rats did not respond to noxious stimuli at this dosage but maintained spontaneous respiration. A direct cut-down was performed on the right groin to expose the femoral artery. The femoral artery was cannulated with a 24-gauge BD angiocath-shielded IV catheter (Becton, Dickinson and Company, Franklin Lakes, NJ, USA), which was then used to withdraw blood, administer drugs, and resuscitate the animals. Continuous hemodynamic monitoring was performed with the Surgivet invasive blood pressure monitor (Med-Electronics, Beltsville, MD, USA). Blood pressure and heart rate measurements were recorded every 2 min. At t = 0 min, rats were hemorrhaged to a mean arterial blood pressure (MAP) of 30 mmHg, which was maintained for 60 min. At the end of the hemorrhage period (t = 60 min), the animals received a bolus injection of warmed sterile phosphate-buffered saline (PBS) (=vehicle, *n* = 4) or bolus injection of 1.8 μg/kg epinephrine in the same volume of warmed sterile PBS (Epi, *n* = 4), followed by fluid resuscitation with PBS at 1.5-times the shed blood volume. To avoid fluid overload, resuscitation was evenly distributed over 30 min (t = 60–90 min) with a maximum bolus injection of 1 mL/min. Sham control animals (sham, *n* = 3) received identical surgical procedures and hemodynamic monitoring without hemorrhage or fluid resuscitation. At t = 90 min, 25 mg/kg of 0.5% Evans Blue (E2219, Sigma-Aldrich) in sterile PBS solution was administered to all animals. At t = 120 min, the animals were euthanized (5% inhaled isoflurane, bilateral pneumothorax via sternotomy). The superior vena cava was occluded, and an 18-gauge peripheral intravenous catheter was introduced into the supradiaphragmatic inferior vena cava (IVC). A total of 20 mL of warmed normal saline was slowly and continuously flushed through the IVC into the right ventricle and pulmonary circulation. The heart and lungs were removed en bloc. Tissue samples were collected from both the right and left lungs. The tissue samples were weighed and used to measure Evans blue concentrations in lung homogenates and to calculate lung wet–dry weight ratios after drying lung samples in an incubator at 70 °C for 24 h.

### 2.7. Measurements of Evans Blue Concentrations

Samples from both lungs were individually homogenized in formamide (1:4 weight/volume) and incubated at 37 °C in a water bath for 24 h. Homogenates were then centrifuged at 250× *g* for 2 min, transferred to a new tube, and centrifuged at 10,000× *g* for 10 min. The absorbance of the supernatant was then measured at 620 nm and 740 nm in a Cytation 1 imaging reader (BioTek Instruments, Winooski, VT, USA). To calculate Evans blue concentrations in the supernatants, absorbance measurements of Evans blue diluted in formamide (0, 5, 10, 15, 20, and 25 μg/mL) were used to generate a standard curve by linear regression analysis (*r*^2^ > 0.99 for all experiments). To correct for heme contamination in the supernatants, the 740 nm measurement was subtracted from the 620 nm measurement [19]. On the basis of the wet–dry weight ratio of each lung sample, Evans blue concentrations were calculated as μg of Evans blue per mg of lung dry weight. 

### 2.8. Data Analyses and Statistics

Data are presented as mean ± standard error (SE). Data were analyzed by 1- or 2-way analyses of variance (ANOVA) with Dunnett’s multiple comparisons tests, as appropriate. Evans blue standard curves were analyzed by linear regression analyses and BRET signals from Gαs activation assays were analyzed by nonlinear regression analyses. All data analyses were calculated with the GraphPad Prism program (Prism 10 for macOS, Version 10.2.3 (347), 21 April 2024). A two-tailed *p* < 0.05 was considered significant.

## 3. Results and Discussion

### 3.1. Adrenoceptor Agonists Improve Normal and Attenuate Thrombin-Induced Impairment of HULEC-5a Barrier Function

**Figure 1:**
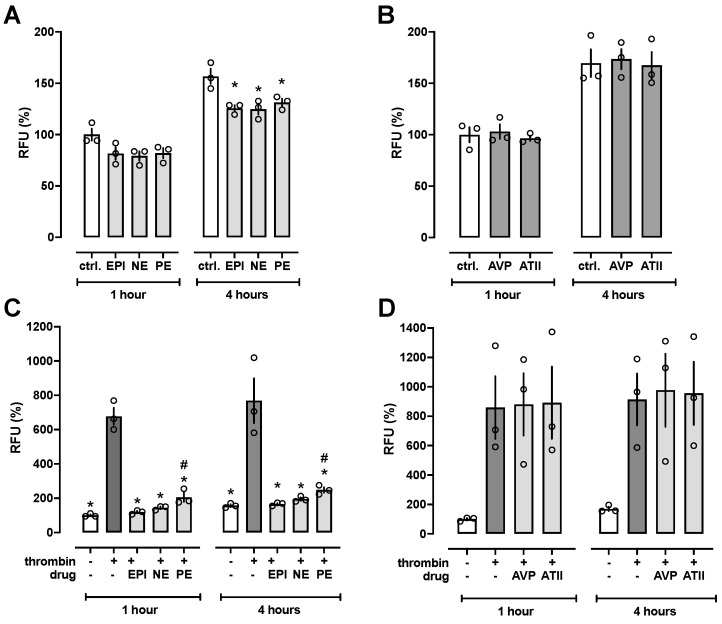
Effects of adrenoceptor agonists, arginine vasopressin (AVP), and angiotensin II (ATII) on normal (**A**/**B**) and thrombin-stimulated (**C**/**D**) HULEC-5a barrier function. RFU (%): Relative fluorescence units (RFU) in % of the RFU measured in untreated cell monolayers after 1 h. Bars and error bars indicate mean ± SE. Open circles show the RFU (%) from duplicate measurements from each experiment. (**A**) HULEC-5a cell monolayers were treated with vehicle (ctrl.) or 5 μM of epinephrine (EPI), norepinephrine (NE), or phenylephrine (PE), and FITC-dextran permeability measured after 1 h and 4 h. *n* = 3 independent experiments. *: *p* < 0.05 vs. vehicle-treated cells after 4 h. (**B**) HULEC-5a cell monolayers were treated with vehicle (ctrl.) or 5 μM of AVP or ATII and FITC-dextran permeability was measured after 1 h and 4 h. *n* = 3 independent experiments. (**C**) HULEC-5a cell monolayers were treated with 25 nM thrombin (+) or vehicle (−) plus vehicle drug (−) or 5 μM of EPI, NE or PE, and FITC-dextran permeability measured after 1 h and 4 h. *n* = 3 independent experiments. *: *p* < 0.05 vs. cells treated with thrombin plus vehicle at the corresponding time point. #: *p* < 0.05 vs. EPI and NE at the corresponding time point. (**D**) HULEC-5a cell monolayers were treated with 25 nM thrombin (+) or vehicle (−) plus vehicle drug (−) or 5 μM AVP or ATII, and FITC-dextran permeability was measured after 1 h and 4 h. *n* = 3 independent experiments.

We performed permeability assays to assess whether adrenoceptor agonists affect HULEC-5a barrier function by monitoring the amount of FITC-dextran that permeated through the HULEC-5a monolayers upon treatment with vehicle or 5 μM of epinephrine (EPI), norepinephrine (NE), or phenylephrine (PE). As shown in Figure 1A, the adrenoceptor agonists reduced FITC-dextran permeability by 18.5–21.4% after 1 h (*p* > 0.05 vs. vehicle) and by 17–21.2% after 4 h (*p* < 0.05 vs. vehicle). The effects of all adrenoceptor agonists were indistinguishable. By contrast, 5 μM AVP or ATII did not affect HULEC-5a permeability (Figure 1B). 

Next, we tested whether adrenoceptor agonists also modulate thrombin-induced impairment of HULEC-5a barrier function (Figure 1C). The treatment of cells with 25 nM thrombin, which is a dose that reflects the EC_50_ concentration of thrombin to induce hyperpermeability [12,13], increased HULEC-5a permeability 6.8-fold after 1 h and 4.9-fold after 4 h. While 5 μM of all adrenoceptor agonists inhibited thrombin-induced hyperpermeability by more than 80% at 1 and 4 h, PE (81.8% and 84.9% inhibition after 1 and 4 h, respectively) was less efficacious than EPI (96.8% and 98.4% inhibition at 1 and 4 h, respectively; *p* < 0.05 vs. PE) and NE (92.3% and 93.6% inhibition at 1 and 4 h, respectively; *p* < 0.05 vs. PE). AVP and ATII (5 μM each), however, did not affect the thrombin-induced impairment of HULEC-5a barrier function (Figure 1D).

### 3.2. β-Adrenoceptor Agonists Attenuate Thrombin-Induced Impairment of HULEC-5a Barrier Function with High Potency

**Figure 2:**
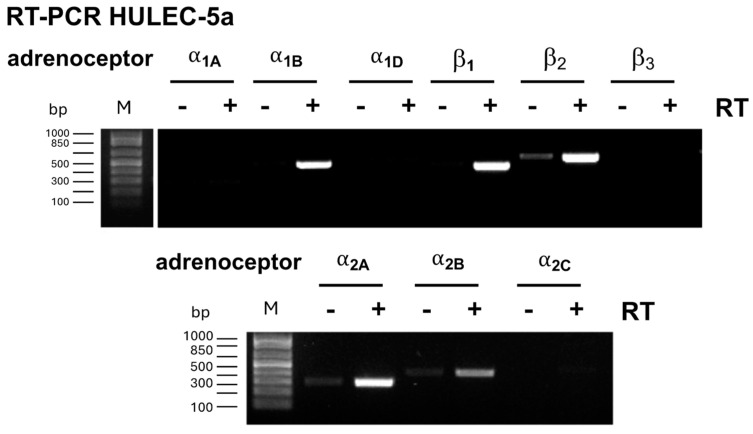
mRNA expression of adrenergic receptors in HULEC-5a cells detected by reverse transcription (RT)-PCR. Images show the agarose gel electrophoresis of cDNAs amplified by PCR and represent *n* = 3 independent experiments. M: molecular marker; RT: reverse transcription.

While EPI and NE activate all adrenoceptors, PE is considered a non-selective α_1_-adrenergic receptor agonist [20]. To evaluate which adrenoceptors mediate the inhibitory effects of their agonists on thrombin-induced barrier function impairment, we performed RT-PCR to assess which adrenoceptors are expressed in HULEC-5a cells. We detected mRNA expression of α_1B_-, α_2A_-, α_2B_-, β_1_-, and β_2_-adrenoceptors, whereas mRNA expression of α_1A_-, α_1D_-, α_2C_-, and β_3_-adrenoceptors could not be observed (Figure 2). 

**Figure 3:**
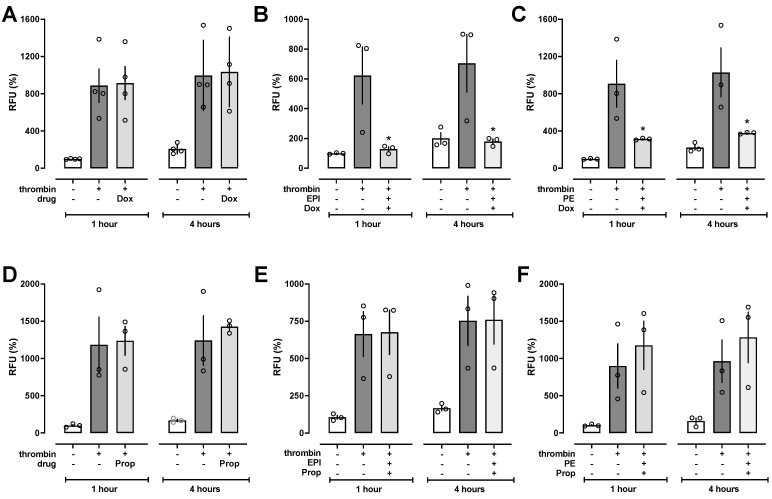
Propranolol but not doxazosin antagonizes the effects of EPI and PE on thrombin-induced impairment of HULEC-5a barrier function. RFU (%): Relative fluorescence units (RFU) in % of the RFU measured in untreated cell monolayers after 1 h. Bars and error bars indicate mean ± SE. Open circles show the RFU (%) from duplicate measurements from each experiment. (**A**/**D**) HULEC-5a cell monolayers were treated with 25 nM thrombin (+) or vehicle (−) plus vehicle drug (−) or 5 μM of doxazosin (Dox, **A**) or propranolol (Prop, **D**) and FITC-dextran permeability measured after 1 h and 4 h. *n* = 3–4 independent experiments in duplicate. (**B**) HULEC-5a cell monolayers were treated with 25 nM thrombin (+) or vehicle (−) plus vehicle drug (−) or 5 μM of doxazosin and 5 μM of EPI, and FITC-dextran permeability measured after 1 h and 4 h. *n* = 3 independent experiments in duplicate. *: *p* < 0.05 vs. cells treated with thrombin plus vehicle. (**C**) HULEC-5a cell monolayers were treated with 25 nM thrombin (+) or vehicle (−) plus vehicle drug (−) or 5 μM of doxazosin and 5 μM of PE, and FITC-dextran permeability measured after 1 h and 4 h. *n* = 3 independent experiments in duplicate. *: *p* < 0.05 vs. cells treated with thrombin plus vehicle. (**E**) HULEC-5a cell monolayers were treated with 25 nM thrombin (+) or vehicle (−) plus vehicle drug (−) or 5 μM of propranolol and 5 μM of EPI, and FITC-dextran permeability measured after 1 h and 4 h. *n* = 3 independent experiments in duplicate. (**F**) HULEC-5a cell monolayers were treated with 25 nM thrombin (+) or vehicle (−) plus vehicle drug (−) or 5 μM of propranolol and 5 μM of PE, and FITC-dextran permeability measured after 1 h and 4 h. *n* = 3 independent experiments in duplicate.

We then tested whether the α_1_-adrenoceptor antagonist doxazosin or the β-adrenoceptor antagonist propranolol inhibits the effects of PE and EPI on thrombin-induced HULEC-5a barrier function impairment. Doxazosin (Figure 3A) and propranolol (Figure 3D) did not affect thrombin-induced hyperpermeability in HULEC-5a cells without adrenoceptor agonists. While doxazosin was unable to block the protective effects of epinephrine (Figure 3B) and phenylephrine (Figure 3C), propranolol abolished the effects of both adrenoceptor agonists (Figure 3E,F).

**Figure 4:**
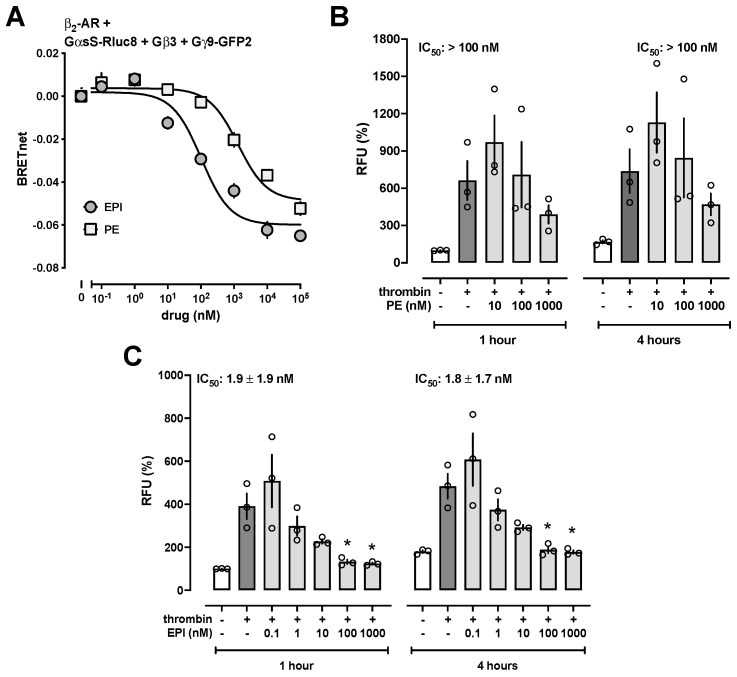
(**A**) β_2_-AR mediated Gαs activation monitored by BRET. HEK293T cells were transfected to express β_2_-AR together with GαsS-Rluc8, Gβ3, and Gγ9-GFP2. Activation of β_2_-AR by epinephrine (EPI) or phenylephrine (PE) leads to dissociation of GαsS from Gγ9 and thereby to the reduction of BRET. Data are the mean ± SE from *n* = 3 independent experiments in duplicate. (**B**/**C**) RFU (%): Relative fluorescence units (RFU) in % of the RFU measured in untreated cell monolayers after 1 h. Bars and error bars indicate mean ± SE. Open circles show the RFU (%) from duplicate measurements from each experiment. (**B**) HULEC-5a cell monolayers were treated with 25 nM thrombin (+) or vehicle (−) in the absence or presence of various concentrations of PE and FITC-dextran permeability measured after 1 h and 4 h. *n* = 3 independent experiments in duplicate. (**C**) HULEC-5a cell monolayers were treated with 25 nM thrombin (+) or vehicle (−) in the absence or presence of various concentrations of EPI and FITC-dextran permeability measured after 1 h and 4 h. *n* = 3 independent experiments in duplicate. *: *p* < 0.05 vs. cells incubated with thrombin plus vehicle.

Because these observations suggest that the effects of EPI and PE are mediated via β-adrenoceptors, we employed TRUPATH BRET biosensors to provide proof of principle that both EPI and PE activate β-adrenoceptor-mediated Gαs activation. These biosensors permit direct observation of G protein activation by monitoring the dissociation of the heterotrimeric Gαβγ complex upon agonist binding to the receptor [15]. Utilizing β_2_-adrenoceptor as an example (Figure 4A), we detected that EPI and PE stimulated β_2_-adrenoceptor-mediated Gαs activation. The potency of PE to activate Gαs, however, was 13-fold lower than the potency of EPI (EPI-EC_50_: 102 ± 29 nM; PE-EC_50_: 1322 ± 369 nM, *p* < 0.01 vs. EPI). Furthermore, the efficacy of PE to activate Gαs (bottom plateau: −0.049 ± 0.003 BRET net) was 20% lower than the efficacy of EPI (bottom plateau: −0.06 ± 0.003 BRET net, *p* = 0.013 vs. PE). These findings align with previous observations suggesting PE may also have β_2_-adrenoceptor agonist activity [21]. 

Consistent with the low potency of PE to stimulate β_2_-adrenoceptor-mediated Gαs activation, we observed in dose-response experiments that PE inhibited thrombin-induced impairment of HULEC-5a barrier function with an IC_50_ of greater than 100 nM (Figure 4B), whereas EPI inhibited thrombin-induced impairment of HULEC-5a barrier function with an IC_50_ of 1.9 ± 1.9 nM and 1.8 ± 1.7 nM after 1 h and 4 h, respectively (Figure 4C). 

Our findings confirm that previous observations on the effects of β-adrenoceptor agonists in the bovine pulmonary artery endothelial cell line CCL 209 in vitro apply to human lung microvascular endothelial cells. This is consistent with observations from in vivo experiments, which showed that propranolol increases normal lung endothelial permeability and that β-adrenoceptor agonists attenuate increases in pulmonary transvascular fluid and protein exchange after thrombin infusion in sheep [4,5,6,7,22]. 

Activation of protease-activated receptor 1 by thrombin is known to impair endothelial permeability via a Ras homolog family member A (RhoA) dependent mechanism [23]. Thus, β-adrenoceptor-mediated activation of the cAMP/protein kinase A pathway, either by inhibition of RhoA activation or via direct stabilizing effects on the endothelial barrier, may contribute to our observations [24,25]. Although protective effects of catecholamines on thrombin-induced barrier function impairment in CCL 209 cells have been attributed to β_2_-adrenoceptor activation [4], selective β_1_- and β_2_-adrenoceptor agonists have been described to protect primary human lung endothelial cell barrier function in other in vitro models [26]. Because we observed that HULEC-5a cells express mRNA for β_1_- and β_2_-adrenoceptors, both β-adrenoceptor subtypes may mediate the protective effects of catecholamines on thrombin-induced barrier function impairment in HULEC-5a cells. The relative contributions of each β-adrenoceptor subtype to the endothelial barrier-protecting effects of catecholamines remain to be determined.

It should be noted, however, that opposite effects of high catecholamine concentrations (5 μM) on normal human umbilical vein endothelial cells have recently been reported [27]. While we cannot exclude that the effects of catecholamines on endothelial cells may depend on the vascular bed from which cells are harvested, permeability-increasing effects of catecholamines were inferred based on measurements of transendothelial electrical resistance [27]. Thus, direct measurements of endothelial cell monolayer permeability appear necessary to confirm these effects. Irrespective of possible differences in endothelial function between different vascular beds, measurements of transendothelial resistance in primary human pulmonary microvascular endothelial cells did not provide evidence that catecholamines reduce transendothelial electrical resistance [26]. In combination with the findings that β-adrenoceptor agonists enhanced transendothelial resistance and prevented decreases in transendothelial resistance induced by the Toll-like receptor agonists in human pulmonary microvascular endothelial cells [26], the findings of the present and previous studies point towards protective effects of catecholamine-stimulated endothelial β-adrenoceptors in the pulmonary circulation under physiological baseline conditions and in disease processes.

### 3.3. Low-Dose EPI Treatment Protects the Lung Vascular Barrier during Resuscitation from Hemorrhagic Shock

To provide proof of principle for such effects of catecholamines in a preclinical animal model, we employed a hemorrhagic shock and fluid resuscitation model in rats to assess whether the in vitro effects of β-adrenoceptor stimulation on lung endothelial cell barrier function translate into lung protective effects in vivo.

**Figure 5:**
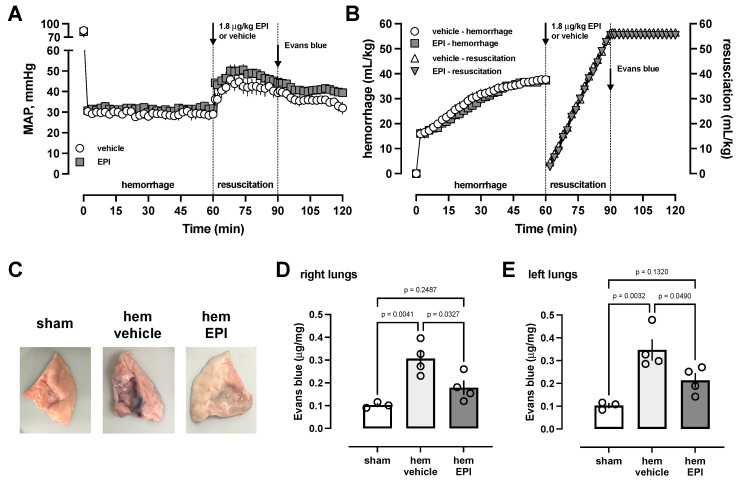
A single low-dose EPI treatment protects the lung vascular barrier during resuscitation from hemorrhagic shock. Animals were hemorrhaged to a MAP of 30 mmHg for 60 min, followed by crystalloid fluid resuscitation with 1.5 times the shed blood volume. At t = 60 min, animals were treated with EPI (grey symbols, *n* = 4) or vehicle (open symbols, *n* = 4). Evans blue was injected at t = 90 min. Animals were euthanized at t = 120 min, and lungs were harvested. Data are the mean ± SE. (**A**) MAP, mmHg. (**B**) Hemorrhage volumes (mL/kg) and fluid resuscitation volumes (mL/kg). (**C**) Typical appearance of lungs from sham-treated animals (left) and animals after hemorrhage and fluid resuscitation with vehicle (hem vehicle, center) or EPI (hem EPI, right) treatment. (**D**/**E**) Quantification of Evans blue extravasation into the right (**D**) and left (**E**) lungs (μg/mg). Sham, *n* = 3. Hem vehicle, *n* = 4. Hem EPI, *n* = 4. Bars and error bars indicate mean ± SE. Open circles show the individual measurements. The levels of statistical significance are shown in the graphs.

The EC_50_ of EPI to inhibit thrombin-induced impairment of HULEC-5a barrier function is in the range of normal baseline catecholamine concentrations in rats [28]. Furthermore, catecholamine concentrations in anesthetized rats have been reported to increase to about 80 nM during hemorrhagic shock [28]. Thus, on the basis of the estimated blood volume in rats [29], we selected a dose of EPI (1.8 μg/kg) that would increase total circulating catecholamine levels at least twofold. In these experiments, we utilized Evans blue extravasation into the lung as a sensitive and quantifiable marker of lung vascular barrier function during resuscitation after hemorrhagic shock [11]. To avoid confounding effects of fluid resuscitation-associated alterations in lung vascular permeability, such as fluid overload, crystalloid fluid resuscitation after hemorrhage was standardized to 1.5 times the shed blood volume. Figure 5A shows the MAPs during the experimental period in animals treated with a single bolus injection of EPI or vehicle at the beginning of the resuscitation period. Figure 5B shows the hemorrhage volumes and the fluid resuscitation volumes. The hemorrhage volumes to achieve a target MAP of 30 mmHg for 60 min and the rates and volumes of fluid resuscitation were indistinguishable between the groups. The injection of 1.8 μg/kg EPI did not result in obvious increases in MAP. This observation is consistent with EPI’s dose–blood pressure response profile previously described in hypotensive rats [30]. 

Furthermore, Evans blue injection did not affect blood pressure. Although MAP was slightly lower in vehicle-treated animals at the end of the observation period, there were no statistically significant differences in MAP between the groups at any time point.

Figure 5C shows representative images of lungs from sham-treated animals, and from animals after hemorrhagic shock and fluid resuscitation with and without EPI injection, and Figure 5D,E the quantification of Evans blue per mg of lung dry weight for the right (Figure 5D) and left (Figure 5E) lungs, respectively. In sham-treated animals, blood pressures were constant throughout the experimental period, and Evans blue injection did not affect the blood pressure. Lungs from sham-treated animals did not show any blue surface staining. Lungs from vehicle-treated animals after hemorrhage and resuscitation showed obvious patches of blue surface staining. By contrast, only minimal or no blue surface staining was visible on the lungs of EPI-treated animals after hemorrhage and resuscitation (Figure 5C). Consistent with these observations, we detected that Evans blue extravasation into the left and right lungs was increased threefold after hemorrhagic shock and fluid resuscitation in vehicle-treated rats as compared with sham-treated rats (Figure 5D,E). Evans blue extravasation into the right and left lungs after hemorrhagic shock and fluid resuscitation in EPI-treated rats was reduced by 63% and 55% (*p* < 0.05 vs. vehicle for both), respectively, when compared with rats after hemorrhagic shock and resuscitation that were treated with vehicle (Figure 5D,E). Evans blue extravasation into the lungs after hemorrhagic shock and fluid resuscitation in EPI-treated rats was not significantly different from rats undergoing sham procedures. 

In conclusion, the findings of the present study demonstrate that β-adrenoceptor agonists, but not arginine vasopressin receptor or angiotensin receptor agonists, enhance normal HULEC-5a barrier function and inhibit thrombin-induced barrier function impairment with high potency and efficacy. Furthermore, we provide proof of principle that a single low-dose EPI injection, which has no relevant effects on blood pressure, at the beginning of fluid resuscitation significantly attenuates hemorrhage and resuscitation-induced impairment of lung vascular barrier function in vivo. Considering that the systemic half-life of EPI is in the range of only a few minutes [31], the finding that these lung-protective effects were detectable 60 min after EPI injection suggests that a short and transient β-adrenoceptor-activation on lung endothelial cells is sufficient to stabilize lung vascular barrier function over periods that far exceed the drug half-life. This starkly contrasts the blood pressure-increasing effects of β-adrenoceptor agonists after single bolus injections, which wear off within seconds to minutes, depending on the administered dose [30]. An obvious limitation of the present model is that it does not permit ARDS or arterial hypoxemia development, even if animals are resuscitated and observed over much longer periods [11,32]. Nevertheless, our findings indicate that hemorrhagic shock and fluid resuscitation lead to a significant impairment of the lung vascular barrier within the early phases of fluid resuscitation, which can be inhibited with single low-dose EPI treatment at the beginning of fluid resuscitation.

While more detailed studies on the dose-effect profile and dosing regimen of EPI after trauma-induced hemorrhage and in other disease processes that impair lung vascular permeability will be required in the future, our findings point toward low-dose β-adrenoceptor agonist treatment as a potential new and promising lung protective strategy to reduce or attenuate development of lung injury and ARDS in patients. Because the effects of EPI occurred at doses that did not result in noticeable effects on blood pressure, such a treatment approach appears to be reasonably safe in patients at risk for ARDS development that do not require vasopressor treatment and is easily translatable into the clinical arena.

## Data Availability

The original contributions presented in the study are included in the article; further inquiries can be directed to the corresponding author.
